# Supplementary material for: Towards deeper understanding of multifaceted chemistry of magnesium alkylperoxides
Source: Commun Chem. 2021 Aug 25;4:123. doi: 10.1038/s42004-021-00560-9 (PMC9814855; doi:10.1038/s42004-021-00560-9)
Supplement: Supplementary file 1 — Description of Additional Supplementary Files. [file 42004_2021_560_MOESM1_ESM.pdf]

Description of Additional Supplementary Files

File Name: Supplementary Data 1

Description: Crystallographic data for the 2_2

File Name: Supplementary Data 1

Description: Crystallographic data for the 3
