# Supplementary material for: Towards deeper understanding of multifaceted chemistry of magnesium alkylperoxides
Source: Commun Chem. 2021 Aug 25;4:123. doi: 10.1038/s42004-021-00560-9 (PMC9814855; doi:10.1038/s42004-021-00560-9)
Supplement: Supplementary file 2 — Supplementary information. [file 42004_2021_560_MOESM2_ESM.pdf]

Towards deeper understanding of multifaceted chemistry of magnesium alkylperoxides

Tomasz Pietrzak[1], Iwona Justyniak[2], Karolina Zelga[1], Krzysztof Nowak,[1] Zbigniew Ochal[1], Janusz Lewiński[1,2]*

[1]Faculty of Chemistry, Warsaw University of Technology, Noakowskiego 3, 00-664 Warsaw (Poland)
[2]Institute of Physical Chemistry, Polish Academy of Sciences, Kasprzaka 44/52, 01-224 Warsaw (Poland)

Table of contents

1. ^{1}H NMR spectra of compounds 1 and 2_2 2
2. X-ray structure determination 3
3. Supplementary References 7

1. ^{1}H NMR spectra of compounds 1 and 2₂

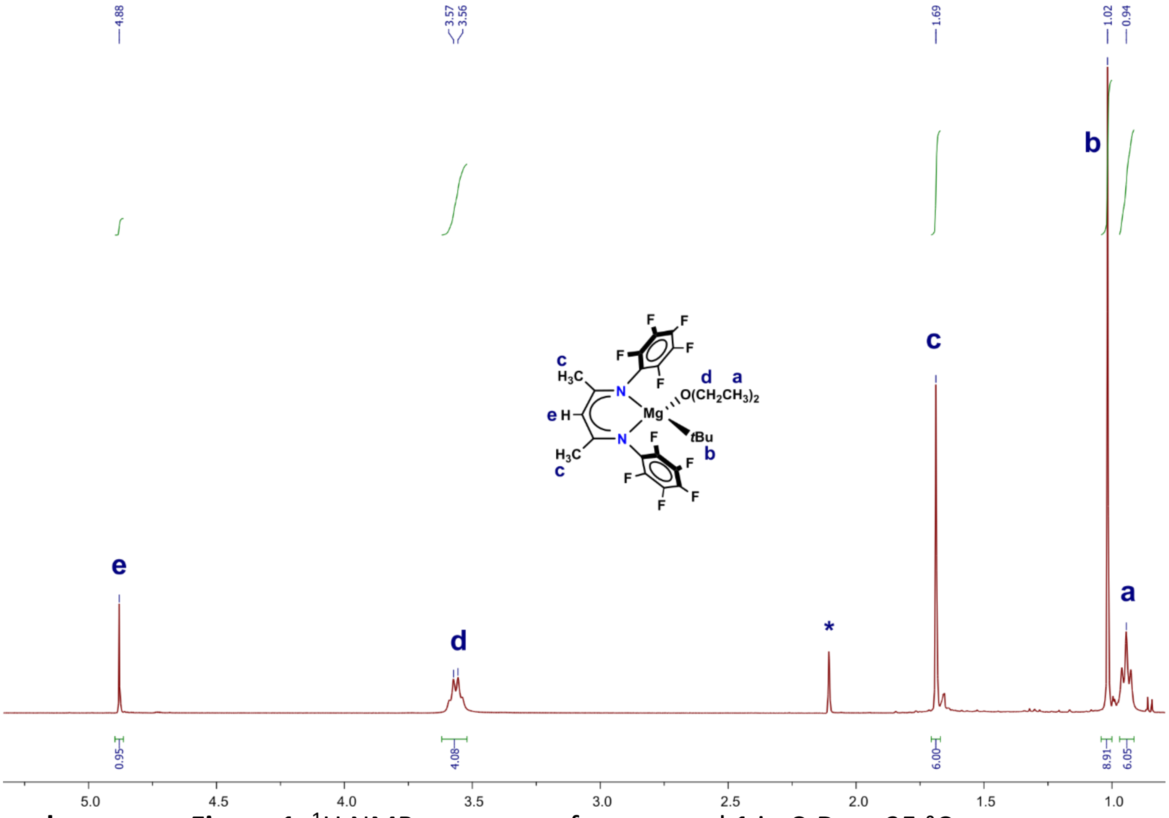

Supplementary Figure 1. ^{1}H NMR spectrum of compound **1** in C_6D_6 at 25 °C.

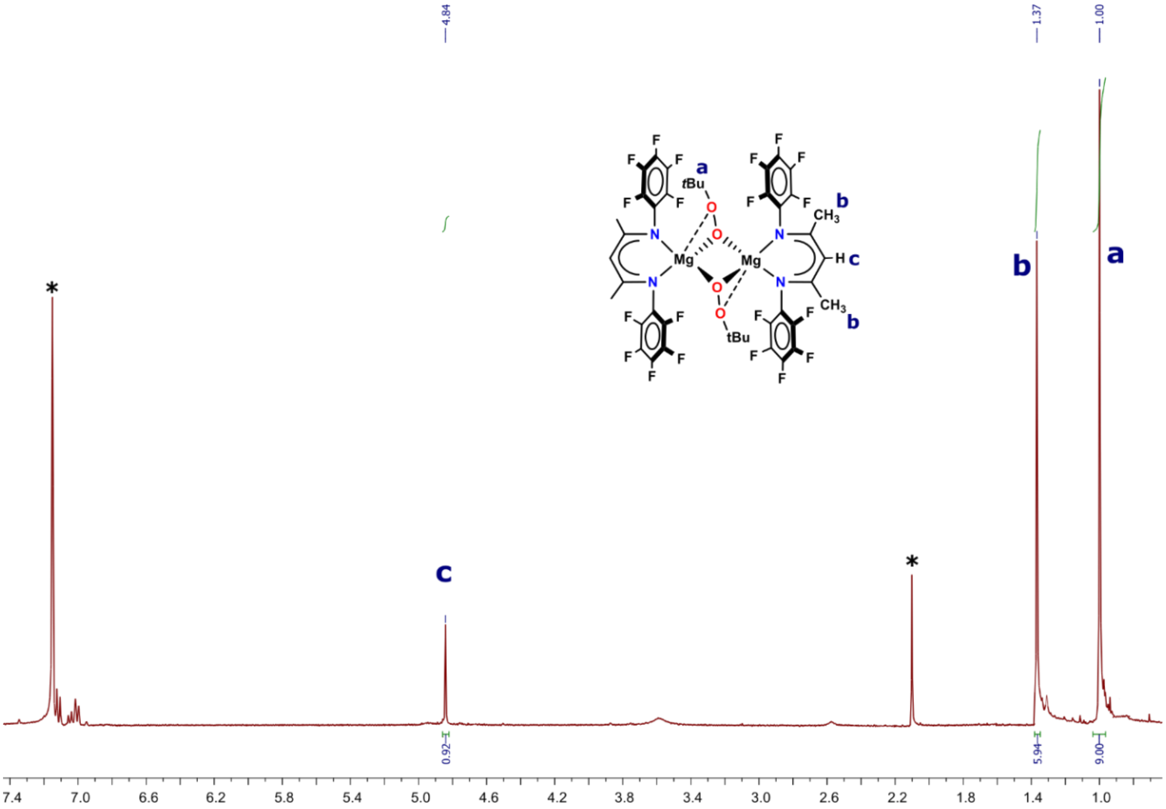

Supplementary Figure 2. ^{1}H NMR spectrum of compound **2₂** in C_6D_6 at 25 °C.

2. X-ray structure determination

The crystals of all complexes were selected under Paratone-N oil, mounted on the nylon loops and positioned in the cold stream on the diffractometer. The X-ray data for complex **2_2** and 3 were collected at 100(2)K on a Nonius KappaCCD diffractometer using Cu*Kα* radiation (λ = 1.54184 Å) and Mo*Kα* radiation (λ = 0.71073 Å) respectively. The data were processed with CrysAlisPro.[1] The structures **2_2** and **3** were solved by direct methods and refined using the SHELXL97.[2] All non-hydrogen atoms were refined with anisotropic displacement parameters. Hydrogen atoms were added to the structure model at geometrically idealized coordinates and refined as riding atoms. Crystallographic data (excluding structure factors) for the structure reported in this paper have been deposited with the Cambridge Crystallographic Data Centre as supplementary publication. Copies of the data can be obtained free of charge on application to CCDC, 12 Union Road, Cambridge CB21EZ, UK (fax: (+44)1223-336-033; e-mail: deposit@ccdc.cam.ac.uk). CCDC(**2_2**)- 2045430, CCDC(**3**)- 2045431.

Supplementary Table 1. Crystal data and structure refinement for 2_2.

Empirical formula	$C_{42}H_{32}F_{20}Mg_2N_4O_4$	
Formula weight	1085.33	
Temperature	100(2) K	
Wavelength	1.54184 Å	
Crystal system	Triclinic	
Space group	P -1	
Unit cell dimensions	a = 9.2134(5) Å	a = 69.428(6)°.
	b = 11.7990(7) Å	b = 77.516(6)°.
	c = 12.0047(9) Å	g = 67.107(5)°.
Volume	1120.77(14) Å^3	
Z	1	
Density (calculated)	1.608 Mg/m^3	
Absorption coefficient	1.685 mm^{-1}	
F(000)	548	
Crystal size	0.18 x 0.12 x 0.08 mm^3	
Theta range for data collection	5.234 to 71.880°.	
Index ranges	-11<=h<=7, -14<=k<=14, -14<=l<=14	
Reflections collected	6764	
Independent reflections	4269 [R(int) = 0.0270]	
Completeness to theta = 67.684°	99.4 %	
Absorption correction	Semi-empirical from equivalents	
Max. and min. transmission	0.874 and 0.785	
Refinement method	Full-matrix least-squares on F^2	
Data / restraints / parameters	4269 / 1 / 330	
Goodness-of-fit on F^2	1.062	
Final R indices [I>2sigma(I)]	R1 = 0.0480, wR2 = 0.1291	

R indices (all data)	R1 = 0.0509, wR2 = 0.1323
Largest diff. peak and hole	0.571 and -0.618 e.Å^{-3}

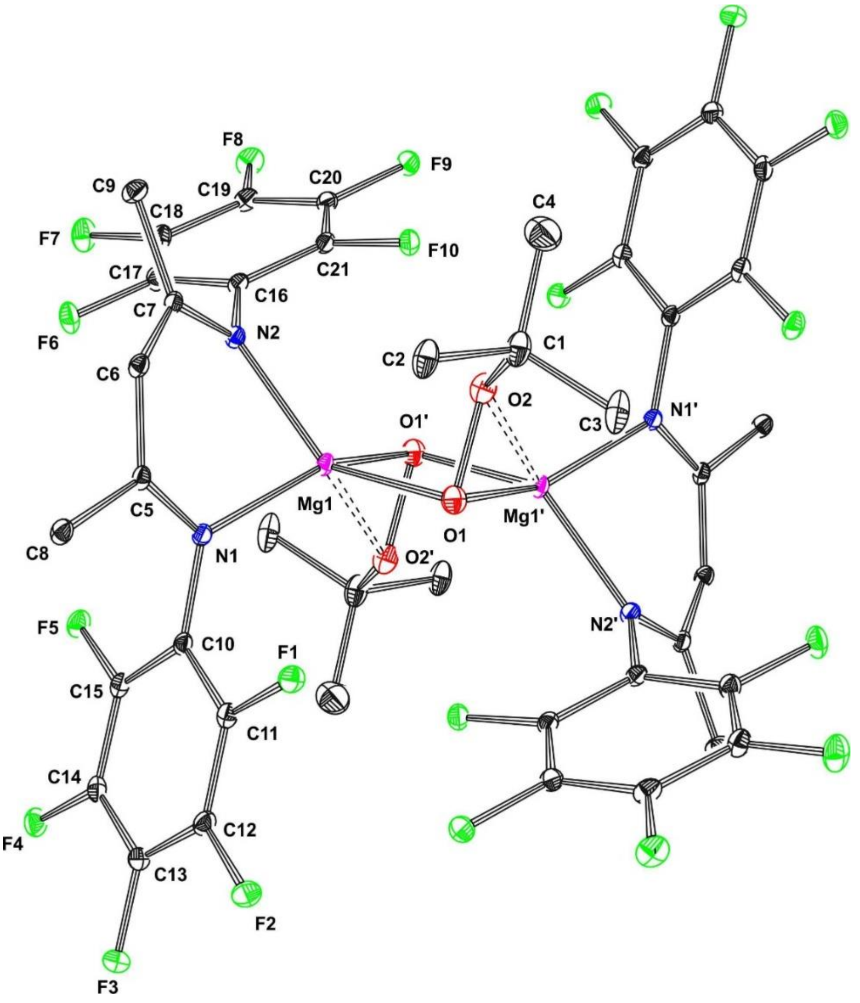

Supplementary Figure 3. Molecular structure of **2**$_2$ with thermal ellipsoids set at 35% probability. Hydrogen atoms have been omitted for clarity. Symmetry transformations used to generate equivalent atoms: (*-x+1, -y+1, -z+1*).

Supplementary Table 2. Selected bond lengths [Å] and angles [°] for **2**$_2$

Mg1-O1	2.0031(16)	Mg1-O1-Mg1’	93.18(6)
Mg1-O1’	2.0256(16)	O2-O1-Mg1	87.65(9)
Mg1-O2’	2.1510(16)	O1-O2-Mg1’	64.71(8)
Mg1-N1	2.0802(17)	O1-Mg1-N1	105.94(7)
Mg1-N2	2.0596(17)	O1’-Mg1-N1	150.94(7)
O1-O2	1.485(2)	O1 – Mg1-N2	135.53(7)
		O1’-Mg1-N2	100.61(7)

	O1-Mg1-O1'	86.82(6)
	N1-Mg1-N2	88.51(6)

Supplementary Table 3. Crystal data and structure refinement for 3.

Empirical formula	$C_{58}H_{68}F_{20}Mg_4N_4O_{12}$	
Formula weight	1490.40	
Temperature	100(2) K	
Wavelength	0.71073 Å	
Crystal system	Triclinic	
Space group	P -1	
Unit cell dimensions	a = 10.0839(4) Å	a = 95.127(4)°.
	b = 11.1700(5) Å	b = 102.809(3)°.
	c = 15.9702(7) Å	g = 104.709(4)°.
Volume	1676.23(13) $Å^3$	
Z	1	
Density (calculated)	1.476 Mg/m^3	
Absorption coefficient	0.171 mm^{-1}	
F(000)	768	
Crystal size	0.19 x 0.14 x 0.07 mm^3	
Theta range for data collection	1.908 to 25.948°.	
Index ranges	-11<=h<=12, -12<=k<=13, -17<=l<=19	
Reflections collected	11018	
Independent reflections	6394 [R(int) = 0.0214]	
Completeness to theta = 25.242°	99.5 %	
Absorption correction	Semi-empirical from equivalents	
Max. and min. transmission	0.988 and 0.972	
Refinement method	Full-matrix least-squares on F^2	
Data / restraints / parameters	6394 / 28 / 453	
Goodness-of-fit on F^2	1.108	
Final R indices [I>2sigma(I)]	R1 = 0.0599, wR2 = 0.1580	
R indices (all data)	R1 = 0.0686, wR2 = 0.1639	
Largest diff. peak and hole	1.039 and -1.128 e.$Å^{-3}$	

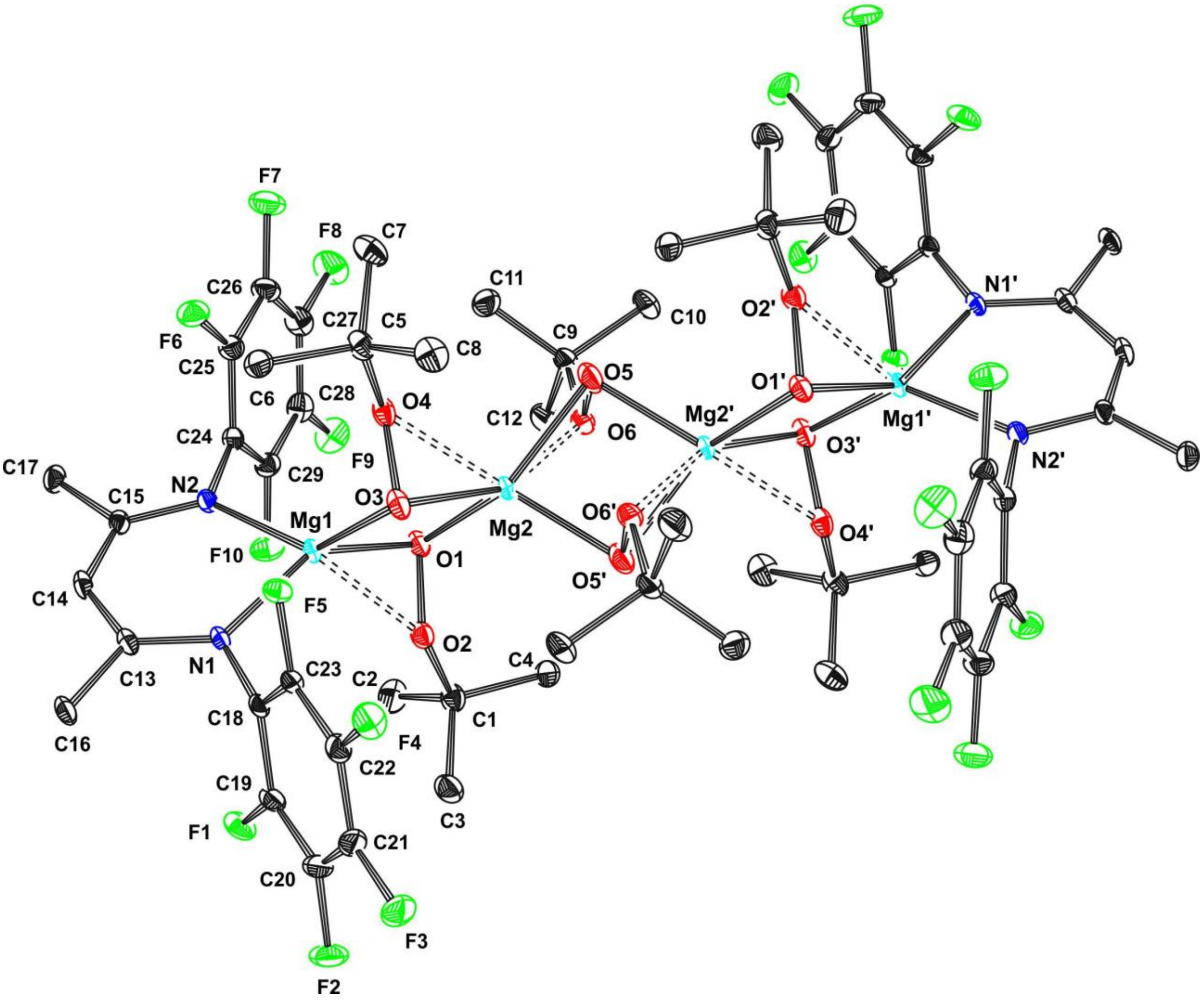

Supplementary Figure 4. Molecular structure of **3** with thermal ellipsoids set at 35% probability. Hydrogen atoms have been omitted for clarity. Symmetry transformations used to generate equivalent atoms: (*-x+2, -y, -z+1*).

Supplementary Table 4. Selected bond lengths [Å] and angles [°] for **3**

Mg1-O1	2.021(2)	Mg1-O1-Mg2	94.20(9)
Mg1-O3	1.983(2)	Mg1-O3-Mg2	95.52(9)
Mg1-O2	2.140(2)	O1-Mg1-N1	145.73(9)
Mg1-N1	2.074(2)	O1-Mg1-N2	103.52(9)
Mg1-N2	2.069(2)	O1–Mg1-O3	85.93(8)
Mg2-O1	2.040(2)	O3-Mg1-N1	104.63(9)
Mg2-O3	2.036(2)	O3-Mg1-N2	137.56(10)
Mg2-O4	2.216(2)	O1-Mg2-O3	84.09(8)
Mg2-O5	2.055(2)	O1-Mg2-O4	85.74(8)
Mg2-O5’	1.990(3)	O1-Mg2-O5	128.28(10)

Mg2-O6	2.139(2)	O1-Mg2-O5’	115.12(10)
O1-O2	1.482(3)	O3-Mg2-O5	129.00(10)
O3-O4	1.454(3)	O3-Mg2-O5’	118.75(10)
O5-O6	1.386(4)	Mg2-O5-Mg2’	94.85(10)
		O5-Mg2-O5’	85.15(10)

3. Supplementary References

[1] Agilent Technologies, *CrysAlisPro*, Version 1.171.35.21b

[2] Sheldrick, G. M. A short history of SHELX. *Acta Crystallogr. Sect. A Found. Crystallogr.* **64**, 112–122 (2008).
